# Tick Cell Culture Analysis of Growth Dynamics and Cellular Tropism of *Rickettsia buchneri*, an Endosymbiont of the Blacklegged Tick, *Ixodes scapularis*

**DOI:** 10.3390/insects12110968

**Published:** 2021-10-27

**Authors:** Cody J. Thorpe, Xin-Ru Wang, Ulrike G. Munderloh, Timothy J. Kurtti

**Affiliations:** Department of Entomology, University of Minnesota, Saint Paul, MN 55108, USA; wang8848@umn.edu (X.-R.W.); munde001@umn.edu (U.G.M.)

**Keywords:** tick cell lines, *Ixodes ricinus* cell line IRE11, blacklegged tick, *Rickettsia buchneri*, *Ixodes scapularis* endosymbiont, RNA FISH, growth dynamics

## Abstract

**Simple Summary:**

The blacklegged tick, *Ixodes scapularis*, a species of significant medical and veterinary importance, harbors an endosymbiont, *Rickettsia buchneri*. This bacterium is largely restricted to the ovaries, but all life stages can harbor different numbers or lack *R. buchneri* entirely. The endosymbiont is cultivable in cell lines isolated from embryos of *Ixodes* ticks. We characterized the cells using microscopy. The doubling time of wildtype *R. buchneri* and a transformant expressing green fluorescent protein was determined to be >7 days when measured by quantitative PCR. Quantification based on fluorescence indicated that 11 days were needed to double the amount of green fluorescent protein. Two rRNA probes were tested using rickettsiae grown in vitro and adapted to localize *R. buchneri* in different organs of field-collected female *I. scapularis* ticks. We observed strong positive signals of *R. buchneri* in the ovaries and surrounding the nucleus of the developing oocytes. The sequestration of rickettsia in ticks and the slow growth dynamics strengthen the contemporary understanding of *R. buchneri* as a transovarially transmitted, non-pathogenic endosymbiont.

**Abstract:**

The blacklegged tick, *Ixodes scapularis*, a species of significant importance to human and animal health, harbors an endosymbiont *Rickettsia buchneri* sensu stricto. The symbiont is largely restricted to the ovaries, but all life stages can harbor various quantities or lack *R. buchneri* entirely. The endosymbiont is cultivable in cell lines isolated from embryos of *Ixodes* ticks. *Rickettsia buchneri* most readily grows and is maintained in the cell line IRE11 from the European tick, *Ixodes ricinus*. The line was characterized by light and electron microscopy and used to analyze the growth dynamics of wildtype and GFPuv-expressing *R. buchneri*. qPCR indicated that the genome copy doubling time in IRE11 was >7 days. Measurements of fluorescence using a plate reader indicated that the amount of green fluorescent protein doubled every 11 days. Two 23S rRNA probes were tested via RNA FISH on rickettsiae grown in vitro and adapted to evaluate the tissue tropism of *R. buchneri* in field-collected female *I. scapularis*. We observed strong positive signals of *R. buchneri* in the ovaries and surrounding the nucleus of the developing oocytes. Tissue tropism in *I. scapularis* and in vitro growth dynamics strengthen the contemporary understanding of *R. buchneri* as a transovarially transmitted, non-pathogenic endosymbiont.

## 1. Introduction and History

One of the most notorious North American ticks from the perspective of human and animal health is the black-legged tick, *Ixodes scapularis*. It is responsible for spreading bacteria that cause Lyme disease (*Borrelia burgdorferi* sensu stricto) and human anaplasmosis (*Anaplasma phagocytophilum*), as well as protozoan blood parasites (*Babesia microti*) and the deer tick lineage of Powassan virus [1,2,3,4], among others. The role of this tick as a major vector of multiple human pathogens spurred the development of *I*. *scapularis* cell lines for the propagation and study of these microbes [5]. *Ixodes scapularis*, a three-host tick, feeds on a large variety of small to large animals, including reptiles [6,7,8], birds, rodents, canids, and deer [9,10,11,12,13,14]. Larvae, nymphs, and females each require a blood meal to develop to the next life-stage or for the production of eggs, providing them a chance to acquire pathogens present in the host’s blood. Competent vectors may subsequently transmit disease agents to a new vertebrate following the molt while taking another blood meal, or pass the agents transovarially to the next generation, as is the case for the genus *Rickettsia*. Given the ability of *I. scapularis* to transmit a wide variety of pathogens, it is puzzling that this tick has not been linked to the transmission of *Rickettsia* species, even though its European counterpart, the sheep tick, *Ixodes ricinus*, is a competent vector of several rickettsial agents, including *Rickettsia monacensis, Rickettsia slovaca,* and *Rickettsia helvetica* [15,16]. Moreover, the range of blacklegged ticks overlaps that of known vectors of *Rickettsia rickettsii*, i.e., the American dog tick, *Dermacentor variabilis*, in areas where Rocky Mountain spotted fever is endemic [17]. This suggests that *I. scapularis* has the opportunity to feed on rickettsiaemic hosts and ingest rickettsiae in the process, which highlights the question of why *I. scapularis* does not seem to transmit pathogens in the genus *Rickettsia*.

While arthropod-borne disease agents of humans and animals have generally garnered most of the attention of researchers, microbes that are symbiotically associated with their invertebrate host have gained increasing attention. Symbionts play multiple roles in the life of their arthropod hosts, recognized as contributing to nutrition as well as to training the immune system to regulate the response to beneficial versus harmful microbes [18,19,20]. Spotted fever-group rickettsiae have consistently been detected in *I. scapularis* ticks of various life stages and have been described as divergent from species known to cause human illness [21,22,23]. Nevertheless, suspicions that these rickettsiae could potentially be human pathogens have lingered. In 2012, Gillespie and colleagues published an analysis of the REIS (rickettsial endosymbiont of *Ixodes scapularis*) genome that revealed extensive disruption of genomic synteny with other genomes in the genus caused by the invasion of mobile elements and concomitant acquisition of genes linked to obligate intracellular parasitism [24]. Altogether, mobile genetic elements and transposases make up over one-third of the genome sequence of this rickettsial species, which includes four plasmids and represents the largest rickettsial genome known. As this sequence was a byproduct of the sequence obtained for the tick host, it was uncertain whether it was complete. The first isolate of this symbiont, named *Rickettsia buchneri,* was obtained and characterized using a cell line, IRE11, isolated from *Ixodes ricinus* [25]. Genes known to be required for infection, intracellular motility, and tolerance to higher temperatures were shown to be defective, corroborating the inability of *R. buchneri* to invade and replicate in mammalian cells. The genome sequence of this isolate revealed two gene clusters missing from the REIS genomic sequence that encode the biosynthetic machinery for the production of antibiotics, not known from other rickettsiae but found in unrelated Gammaproteobacteria [26]. This ignited new interest in the relationship between *R. buchneri,* its tick host, and the pathogens transmitted by *I. scapularis*. Although previous research has demonstrated rickettsiae in the ovaries of *I. scapularis* [27], and it is known that *R. buchneri* persists transovarially and transstadially in ticks [28,29], its distribution in tick tissues is incompletely understood.

Tick cell lines provide powerful systems for propagating and determining the relationships between beneficial endosymbionts and their pathogenic relatives [25,30,31,32]. Here, we describe tick cell lines that we used to characterize *R. buchneri* and analyze its interaction with host cells. We present the growth and cytopathic features that set *R. buchneri* apart from known pathogenic rickettsiae. We also describe the application of an *I. ricinus* cell line to develop a fluorescent RNA probe to map *R. buchneri* in different organs and cells of female blacklegged ticks. The proximity of *R. buchneri* and tick-borne pathogens in key organs may explain the ability or inability of ticks to maintain and transmit these pathogens.

## 2. Materials and Methods

### 2.1. Tick Cell Lines

Cell lines isolated from embryos of *Ixodes ricinus* (IRE11) and *Ixodes scapularis* (ISE6 and ISE18) [5,33] were cultured in an L15C300 medium [34] supplemented with fetal bovine serum (FBS, 5%), tryptose phosphate broth (TPB, 5%; Difco, Franklin Lakes, NJ, USA), and lipoprotein concentrate (LPC, 0.1%; MP Biomedicals, Irvine, CA, USA), hereafter referred to as the complete medium. Uninfected cultures maintained at 32 to 34 °C had population doubling times of 4 to 5 days and were subcultured every 2 to 4 weeks by splitting cultures with 1 to 5 or 10 confluent cell layers [5]. At 26 to 28 °C, cells remained adherent but grew slowly, with doubling times of 2 to 3 weeks. The medium was changed weekly, regardless of the incubation temperature or cell density. We confirmed the species identity of the lines by PCR sequencing the mitochondrial 16S rRNA gene [35,36].

### 2.2. Rickettsia buchneri

*Rickettsia buchneri* (ISO7^T^), isolated from ovaries of an *I. scapularis* female [25], was grown in IRE11 cells. An ISO7 transformant expressing GFPuv [37] was also maintained in IRE11. The subline was transformed with the shuttle vector pRAM18dRGA derived from pRAM18 of *Rickettsia amblyommatis* [38]. The vector carries a selection cassette encoding the *Rickettsia prowazekii arr*-2 rifampin resistance gene (*rpsLp-arr-2Rp*) and a reporter gene encoding a green fluorescent protein (GFPuv).

Both strains were subcultured every five to seven weeks by mixing 1 mL (−2 × 10^6^ cells) of infected cells with 4 mL of uninfected IRE11 cells (−10^7^ cells) in 25 cm^2^ flasks [25,37] using the complete medium. Infection levels (percentage of cells infected and quantity of rickettsiae per cell) were determined using Giemsa-stained cell preparations (see Section 2.4). Cultures containing cells infected with fluorescent *R. buchneri* were quantified by fluorescence microscopy (see Section 2.4). Cells in infected cultures adhered loosely and were fed weekly by centrifuging the cells (360 rcf, 6 min, 12 °C), and cell-free *R. buchneri* in the supernatant were discarded. The cell pellet was resuspended in a fresh medium and transferred back to the original flask. Cultures were maintained in ambient air and incubated at 26–28 °C. Initially, subcultures contained 20–25% infected cells, and when infection levels reached >90% subcultures were made [25]. *Rickettsia buchneri* numbers per cell reached high levels, and cells contained more than 100 rickettsiae per cell (as determined by Giemsa stain).

### 2.3. Challenge of Ixodes Cell Lines with Cell-Free R. buchneri

Cell-free *R. buchneri* was prepared following protocols previously published for *Rickettsia peacockii* [39]. Briefly, cells were lysed using shear forces and the suspension was filtered through a 2 µm pore-size filter and concentrated by centrifugation. We quantified cell-free *R. buchneri* harvested from 25 cm^2^ flasks of heavily infected IRE11 cells by using a Petroff–Hausser bacterial counting chamber. To estimate infectivity (multiplicity of infection, MOI), serial 10-fold dilutions of the rickettsial suspension (10^8^–10 rickettsiae) were inoculated into wells of a 24-well plate containing 1 × 10^6^ uninfected IRE11 cells/well (MOI 10^2^–10^−5^). Pelleted *R. buchneri* were resuspended and centrifuged onto IRE11, ISE6, and ISE18 cell layers cultured at 28 °C in 24-well plates placed in a candle jar [40].

### 2.4. Microscopy of Cells Infected with Rickettsia buchneri

*Phase contrast microscopy*: We examined living cultures for infected cells and cell-free rickettsia with an inverted phase contrast microscope (Diaphot, Nikon Instruments Inc., Melville, NY, USA) fitted with 20× and 40× objectives.

*Giemsa stain*: Cytocentrifuge preparations of IRE11 cells were prepared, usually at the time of a medium change or subculture, using a Cytospin 4 (Thermo Fisher Scientific, Waltham, MA, USA) cytocentrifuge. Cells were centrifuged at 113 rcf onto glass microscope slides, air dried, fixed in absolute methanol, and stained with Giemsa stain (4% in Sorenson’s buffer, pH 6.6). Infection levels (percent cells infected) were estimated by light microscopy. Infection levels (percentage of cells infected and the approximate number of rickettsiae per cell) were determined by Giemsa-staining cytocentrifuge smears.

*Fluorescence microscopy of GFP expressing R. buchneri*: Images of GFPuv-*R. buchneri* in IRE11 cells were taken using a Nikon Eclipse E400 fluorescence microscope fitted with fluorescein isothiocyanate (FITC) filters (Dil/Cy3 (Chroma Technology, Bellows Falls, VT, USA). Digital images of a drop of cell suspension under a coverslip were acquired with a DMX-1200 digital camera. An inverted microscope (Nikon Diaphot fitted with a sapphire GFP filter) was used to examine live cultures containing cells infected with fluorescent *R. buchneri*. Infection levels (percentage of cells infected and the approximate number of rickettsiae per cell) were estimated via fluorescence microscopy.

*Transmission Electron Microscopy*: Uninfected and infected IRE11 cells were prepared for transmission electron microscopy as described previously [41,42,43]. Briefly, cells were fixed in glutaraldehyde and post-fixed in osmium tetroxide, dehydrated, infiltrated, embedded, and sectioned using standard methods. Stained sections were examined using a Philips CM12 transmission electron microscope.

### 2.5. R. buchneri In Vitro Growth Dynamics: qPCR Analysis

We determined the growth dynamics of wildtype- and GFPuv-transformed *R. buchneri* in IRE11 cells grown in 24-well culture plates. Infected cells from 25 cm^2^ flasks were centrifuged (12 °C; 360 rcf; 6 min) and the supernatant containing cell-free rickettsiae was discarded. Cell pellets were resuspended in a fresh complete medium and diluted to 3 × 10^6^ cells/mL and inoculated into clear 24-well flat bottom tissue culture plates (1 mL of infected cells per well). Initial infection levels were determined by Giemsa-staining cytocentrifuge smears of inocula (see Section 2.4). Water was added to spaces bordering wells to prevent dehydration. Plates were incubated at 26 °C in a humidified candle jar [40]. At selected times (0, 48 h, 96 h, and 168 h), the contents of 3 replicate wells were transferred to microfuge tubes and centrifuged (13,600 rcf for 5 min). DNA was extracted from cell pellets using QIAGEN Puregene Core Kit A (QIAGEN, Valencia, CA, USA) following the protocol for purification from Gram-negative bacteria and eluted in a final volume of 50 µL of a DNA hydration solution (10 mM Tris, 1 mM ethylenediaminetetraacetic acid [EDTA], pH 7–8). The DNA concentration was measured using a DeNovix DS-11 FX Spectrophotometer-Fluorometer (DeNovix, Wilmington, DE, USA) and the solution was diluted 1:400 for qPCR analysis to determine the *R. buchneri* citrate synthase (*gltA*) gene copy number following the protocol given by Oliver et al. [29]. The primer sets CS-F (5′-TCGCAAATGTTCACGGTACTTT-3′) and CS-R (5′-TCGTGCATTTCTTTCCATTGTG -3′) (DNA Technologies; IDT, Coralville, IA) were used to amplify rickettsial *gltA* gene copies [44]. *Rickettsia buchneri gltA* copy numbers were calculated utilizing the molecular weight of the plasmid standard. Data were plotted and analyzed using GraphPad Prism version 9.1.2 for macOS.

### 2.6. Fluorescence Plate Reader Analysis of R. buchneri Growth

Suspensions of IRE11 cells infected with transformed *R. buchneri* were adjusted to concentrations of 1.5 × 10^6^ cells/mL and 3 × 10^6^ cells/mL. Initial infection levels were determined by Giemsa-staining cytocentrifuge smears of inocula (see Section 2.4) and by routine examination of the wells by fluorescence microscopy (see Section 2.4). Infected cells were seeded in triplicate into 96-well, black-walled, clear-bottomed tissue culture plates at 200 µL per well with a water border of 200 µL per well surrounding all treatment wells to prevent desiccation. Additionally, uninfected IRE11 cells, adjusted to the same concentration standard of 3 × 10^6^ cells/mL, were seeded in triplicate at a volume of 200 µL per well to measure the background fluorescent signal from IRE11 host cells. Plates were incubated in a humidified candle jar inside a 30 °C incubator for 24 h to encourage cell adherence to the bottom of the plate wells. Plates were then read, without shaking, for total fluorescent units of GFPuv (excitation: 395 nm and emission: 509 nm) on a Biotek Synergy H1 Hybrid Plate Reader (Biotek, Winooski, VT, USA) set to an incubation temperature of 30 °C. Data were collected by using Gen5 v. 3.08 software (Biotek, Winooski, VT, USA). After the initial 24 h reading, the plates were sealed and returned to the 30 °C incubator inside the humidified desiccator for another 168 h and 312 h. Fluorescence was subsequently measured following the same protocol. To calculate total fluorescent units solely produced by transformed *R. buchneri*, the fluorescent signal from uninfected IRE11 cells was subtracted from the fluorescent signal produced by transformed *R. buchneri* from each plate.

### 2.7. RNA FISH: 23S rRNA Fast Red-Labeled Rickettsial Probe Design

To localize intracellular *R. buchneri,* a FISH assay was developed. RNA fluorescent in situ hybridization (FISH) utilizing the ViewRNA Tissue Assay Core Kit from Thermo Fisher Scientific (Waltham, MA, USA) was used. A rickettsial-specific DNA probe set (ATATTCACTGCACAATCAATCCAA and TGAAGGTGTTATTTCCTGGGAA) was designed utilizing the 23S rRNA gene sequence from *R. buchneri* (*Rickettsia buchneri* strain ISO7 contig 080 whole genome shotgun sequence: Genbank Accession Number: JFKF01000080.1) [43].

### 2.8. In Vitro RNA FISH Assay

The efficacy of the 23S Fast Red-labeled rickettsial probe was tested with *R. buchneri* grown in IRE11 cells using a modified protocol based on the ViewRNA Tissue Assay Core Kit protocol, as described in [43]. IRE11 cells containing wildtype or transformed *R. buchneri* were used to test the 23S probe. Uninfected and heavily infected cells (90% infected as determined by Giemsa staining) were centrifuged onto microscope slides using a Cytospin 4 centrifuge (Thermo Fisher Scientific, Waltham, MA, USA) and fixed using neutral buffered formalin (NBF) (10%, 100 µL) at 4 °C for 1.5–2 h. To expose the 23S genes and allow penetration of the probe into the cell, fixed cells were pretreated with AQUEOUS Triton X-100 (Sigma-Aldrich, St. Louis, MO, USA) (100 µL of 1:1000) for 2 h at room temperature. After permeabilization, we followed the instructions for the ViewRNA Tissue Assay Core Kit from Thermo Fisher Scientific (Waltham, MA, USA). Slides were mounted in the ProLong Diamond Antifade Mountant with 4′,6-diamidino-2-phenylindole (DAPI; Thermo Fisher Scientific; Waltham, MA, USA), and overlaid with cover slips. Nail polish was utilized to seal the edges of the cover slip to aid in long-term storage and to prevent desiccation. Slides were stored in a humid chamber at 4 °C until being examined with an Olympus Disc Scanning Unit (DSU) confocal microscope (Olympus, Shinjuku City, Tokyo, Japan) (60× objective). The following excitation and emission parameters used for fluorescent imaging were 4′,6-diamidino-2-phenylindole (DAPI); excitation 350 nm, emission 470 nm; tetramethylrhodamine (TRITC) excitation 557 nm, emission 576 nm. Raw data images were edited with ImageJ Fiji software [45].

### 2.9. Tick Collection and Maintenance

Flat (unfed) *I. scapularis* females were collected at Camp Ripley, MN (46.1661° N, 94.3603° W) from hunter-killed white-tailed deer. Ticks were briefly sanitized in a 1:50 dilution of bleach followed by a sterile water rinse. Ticks were dried on sterile filter paper, transferred to sterile cell strainers (Corning Falcon, Tewksbury, MA, USA), and placed in sealed desiccator jars containing a saturated solution of K_2_SO_4_ (RH > 95%) and held in a tick rearing room (16:8 L:D, 22 °C).

### 2.10. RNA FISH Assay Dissected Whole Organs

The single-plex 23S Fast Red rickettsial probe was used to evaluate the tissue tropism of *R. buchneri* in organs (ovaries, salivary glands, seminal receptacles, midguts, and synganglia) of flat *Ixodes scapularis* females. Organs were dissected and immediately placed in a 1.5 mL centrifuge tube containing 10% NBF and fixed for 3–7 d at 4 °C. Organs were repeatedly washed in PBS. If needed, pooled organs were gently centrifuged at 100 rpm for 1 min to ensure organs had moved to the bottom of the tube. A 1:1000 dilution of Triton X-100 (Sigma-Aldrich, St. Louis, MO, USA) was used to permeabilize whole organs for 3 h.

Whole-organ mounts were prepared on microscope slides with the ProLong Diamond Antifade Mountant with DAPI (Thermo Fisher Scientific; Waltham, MA, USA) and additionally, the coverslip was sealed with nail polish to prevent desiccation during long-term storage. Whole organ mounts were imaged at 4×, 10×, and 60× objective magnification at the University of Minnesota Imaging Center with a Nikon A1 Spectral confocal microscope (Nikon, Minato City, Tokyo, Japan). The following excitation and emission parameters were utilized for fluorescent imaging: 4′,6-diamidino-2-phenylindole (DAPI): excitation 350 nm, emission 470 nm; Tetramethylrhodamine (TRITC): excitation 557 nm, emission 576 nm (Thermo Fisher Scientific, Waltham, MA, USA). Raw data images were edited with ImageJ Fiji software [45].

## 3. Results

### 3.1. Challenge of Ixodes Cell Lines with R. buchneri

The ISO7 strain of *R. buchneri* was isolated from ovarian tissues of a partially engorged adult *I. scapularis* [25]. This strain has been maintained in *I. ricinus* IRE11 cells since 2007 and has been serially transferred >50 times by mixing infected cells with uninfected cells. Infected cells were not active mitotically and it was necessary to add uninfected cells at each transfer at a ratio of 1:5 or 1:10. When cell preparations were examined using Giemsa staining, initially 10–20% of the cells were infected and the infectious inoculum harbored highly variable numbers (10 to >100) of *R. buchneri* per cell. Mixing infected IRE11 cells with the *I. scapularis* lines ISE6 and ISE18 did not result in the establishment of *R. buchneri* in these lines.

**Figure 1:**
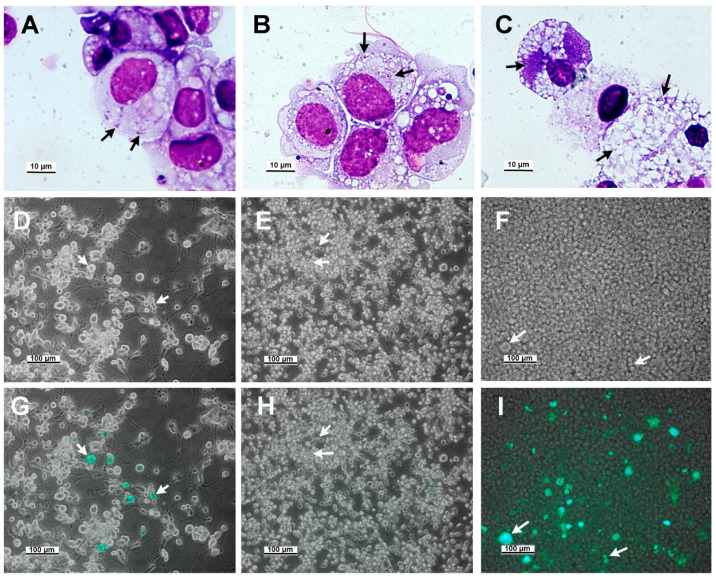
*Ixodes scapularis* ISE6 and ISE18 cell lines and *I. ricinus* cell line IRE11 challenged with cell-free *R. buchneri*. (**A**–**C**) Giemsa staining of cells deposited onto microscope slides by centrifugation, day 15 post inoculation (pi). (**A**) ISE6 cell day 15 pi. (**B**) ISE18 cell day 15 pi. (**C**) IRE11 cell day 15 pi. (**D**–**F**) Phase-contrast microscopic image of lines 42 days pi; ISE6 (**D**), ISE18 (**E**), and IRE11(**F**) 42 days pi of cell-free *R. buchneri*. (**G**–**I**) Fluorescence microscopic images merged onto phase-contrast images in panels (**G**–**I**). Arrows point to selected cytoplasmic rickettsiae or cells rickettsiae (**A**–**C**) or cells containing fluorescent rickettsiae.

We compared the infectivity of cell-free GFPuv *R. buchneri* for ISE6, ISE18, and IRE11 using Giemsa staining of fixed cell cytospin smears 14 days post inoculation (pi) (Figure 1A–C) and phase contrast–fluorescence microscopy of living cultures 42 days pi (Figure 1D–I). The centrifugation of cell-free *R. buchneri* onto ISE6 and ISE18 cells resulted in low infection levels (percent of cells infected and the number of rickettsiae per cell) (Figure 1A,B,D–G). In contrast, *R. buchneri* established robust infections in IRE11 cells (Figure 1C). Our attempts to establish subcultural infections of ISO7 in *I. scapularis* cell lines ISE6 and ISE18 using cell-free rickettsiae have been unsuccessful.

### 3.2. Microscopic Features of R. buchneri Growth in the Ixodes ricinus Cell Line IRE11

**Figure 2:**
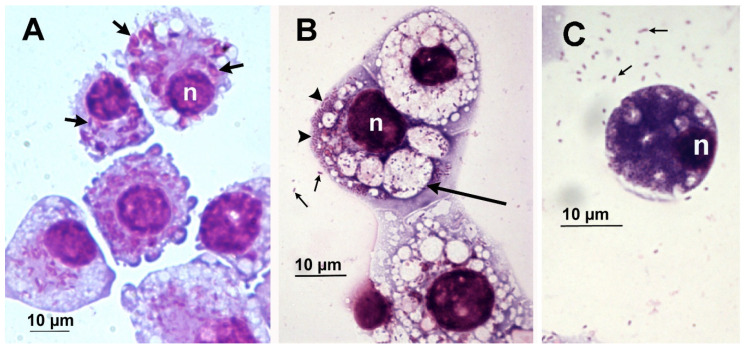
*Ixodes ricinus* cell line IRE11. Giemsa staining of cells deposited onto microscope slides by centrifugation. (**A**) Uninfected IRE11 cells in passage 89. Note characteristic mauve-colored ellipsoidal bodies present in cytoplasm of most cells (short arrows). (**B**) IRE11 cells (passage 45) infected with *Rickettsia buchneri* (at passage 29). (**C**) IRE11 cell heavily infected with *R. buchneri.* Long arrows point to vacuoles with rickettsiae. Big arrowheads point to cytoplasmic clusters of rickettsiae while small arrows point to cell free rickettsiae. Nuclei denoted with n.

**Figure 3:**
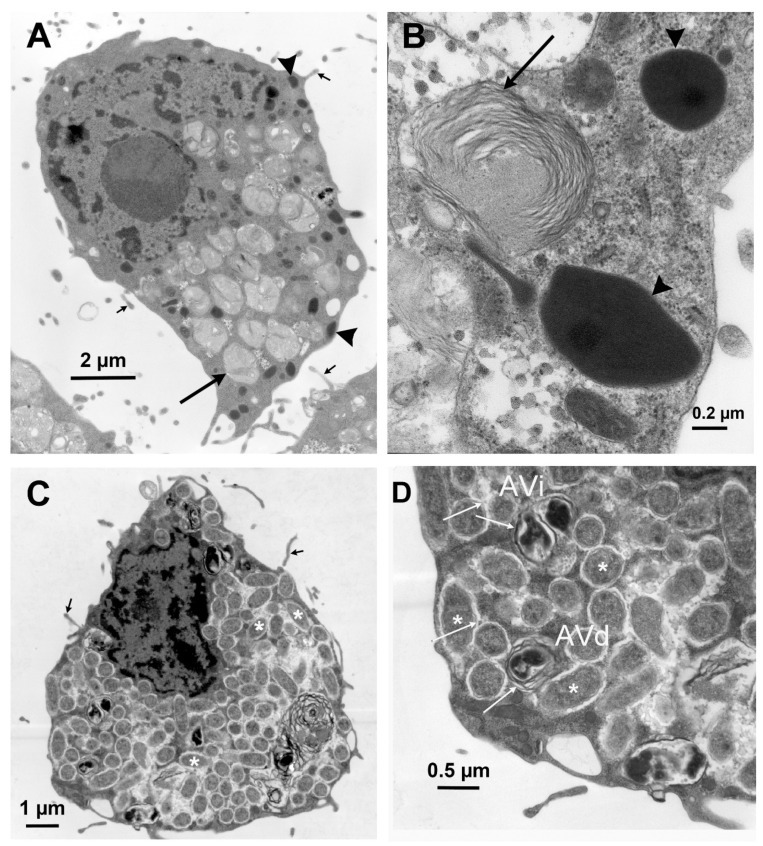
Transmission electron micrographs of *Ixodes ricinus* cell line IRE11. (**A**) Uninfected IRE11 cell. Cells contain numerous vacuoles, possibly representing secondary lysosomes with indigestible residual bodies (large arrows) and electron dense ellipsoidal inclusions (arrowheads). (**B**) Cytoplasmic inclusions containing layers of fibrillary structures (arrow) and electron dense bodies (Arrowhead) found in uninfected IRE11 cells. (**C**) IRE11 cell infected with *R. buchneri* (white asterisks). Small arrows in (**A**,**C**) point to filopodia. (**D**) Enlarged portion of (**C**) (lower left corner). White arrows point to *R. buchneri* within autophagosomes (AVi and AVd).

In the IRE11cell line, *Rickettsia buchneri* grew intracytoplasmically, often within vacuoles (Figure 1 and Figure 2). IRE11 cells typically contain ellipsoidal granules that stain mauve with Giemsa staining (Figure 2A) and are electron dense in electron micrographs (Figure 3). Uninfected IRE11 cells contain numerous circular structures containing membranes; these cellular structures are reduced in heavily infected cells (Figure 2C and Figure 3C,D). Filipodia are present in uninfected and infected cells (Figure 3C,D). In addition, autophagosomes were observed in infected cells. These double-membrane-bound vacuoles contained *R. buchneri* in different stages of degradation. Within initial autophagosomes (AVi), individual *R. buchenri* were surrounded by a double membrane, while multi-membrane structures were formed within degraded autophagosomes (AVd) (Figure 3D).

**Figure 4:**
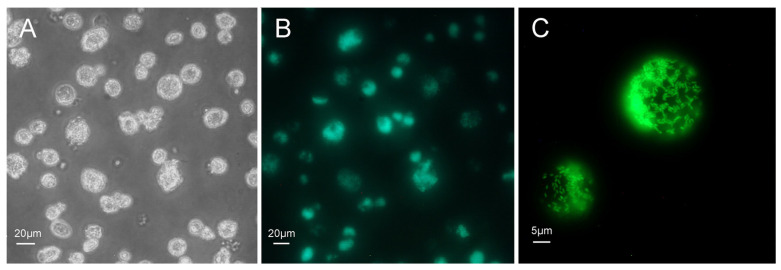
*Ixodes ricinus* cell line IRE11 infected with *Rickettsia buchneri* transformed to express GFPuv. (**A**) Phase-contrast microscopy image of infected IRE11 cells. Cells were diluted to permit imaging of individual IRE11 cells and intracellular *R. buchneri* expressing GFPuv. (**B**) Fluorescence microscopy image of cells shown in panel (**A**). Note variable levels of infection among IRE11 cells and that the infection level is 80%. (**C**) High magnification fluorescence microscopy image of two infected IRE11 cells.

We also tracked the in vitro growth of *R. buchneri* with a transformant that expressed GFPuv. Live cell imaging was used to estimate the proportion of infected cells and visualize heavily infected cells (Figure 4). We estimated infection levels in individual cells by comparing phase-contrast and fluorescence images (Figure 4A,B). Largely infected cells were hypertrophied and contained large numbers of rickettsiae (Figure 4C).

While Giemsa staining or fluorescence microscopy allowed us to measure the proportion of infected cells, microscopy alone did not permit accurate estimation of *R. buchneri* population size. We used qPCR targeting the single-copy gene *glt* to quantify the number of rickettsiae in heavily infected cultures, assuming that each rickettsia contains a single copy of the genome.

### 3.3. Growth Dynamics of R. buchneri in IRE11 Cells by qPCR

**Figure 5:**
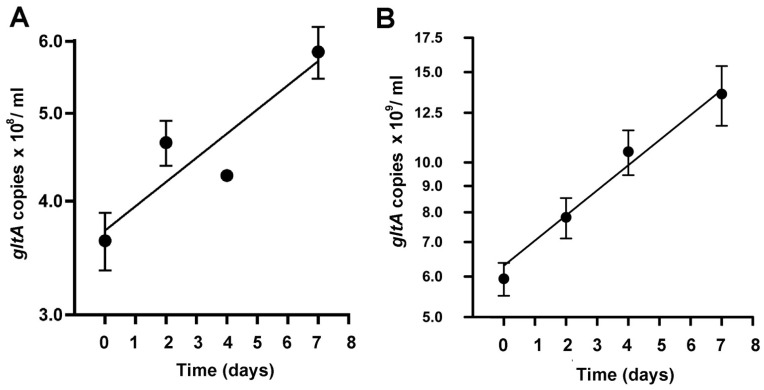
In vitro growth curve of (**A**) wildtype and (**B**) GFPuv-*R. buchneri*. Heavily infected IRE11 cells were grown at 26 °C. Growth was estimated by quantifying citrate synthase (*glt*), a single-copy gene, over time by qPCR. The Y axes indicate the number of genome (*glt*) copies per 3 × 10^6^ IREll cells. Error Bars indicate SEM; n = 3. Y-axes scales are log_2_.

We used the rickettsial-specific single-copy gene, *gltA* to estimate the number of *R. buchneri* in heavily infected IRE11 cells [29,44,45]. The number of *gltA* copies obtained from wells inoculated with 3 × 10^6^ infected cells was used to measure the genome copy number per ml. The infection rate (percentage of infected cells) was greater than 90% as determined by Giemsa staining. In heavily infected cultures, *R. buchneri* grew slowly at the rate of 10 (wildtype) and 7 (GFPuv) days per genome copy doubling (Figure 5) with approximately 100 to 1000 rickettsiae per cell.

### 3.4. Growth Dynamics of R. buchneri in IRE11 Cells under Elevated Temperature Stress

**Figure 6:**
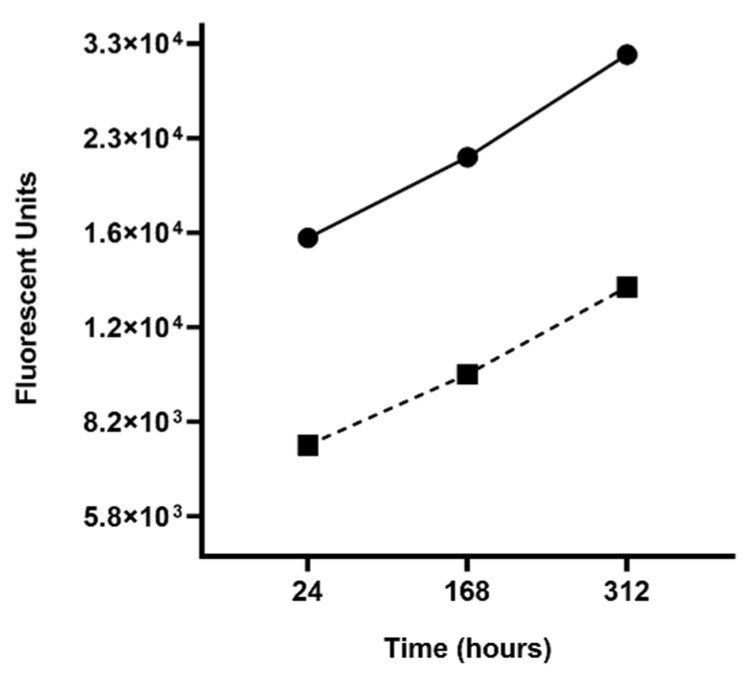
Growth curve of GFPuv-transformant *R. buchneri* grown at 30 °C in IRE11 cells measured with a fluorescence plate reader (excitation: 395 nm and emission: 509 nm). Solid line represents 3 × 10^6^ cells/mL; dashed line depicts growth of cells at 1.5 × 10^6^ cells/mL. Error bars indicate SEM; n = 6. Y-axis is log_2_ scale.

The genome sequence of *R. buchneri* ISO7 revealed the disruption of genes involved in dealing with heat shock (*hsp* genes; [25]). To test the sensitivity of this *R. buchneri* isolate to an elevated temperature of 30 °C, we used a fluorescence plate reader to measure GFPuv expression as a proxy for *R. buchneri* numbers and growth. The fluorescence plate reader efficiently quantified IRE11 cells infected with GFP_UV_-expressing *R. buchneri* at two different concentrations (3 × 10^6^ cells/mL and 1.5 × 10^6^ cells/mL) over time. Both cell concentrations grew consistently relative to each other over the course of approximately 2 weeks (Figure 6), indicating that in wells seeded with fewer cells, growth proceeded at the same rate as in those containing twice as many cells. The doubling time for GFP_UV_-expressing *R. buchneri* was approximately 11 days.

### 3.5. 23S rRNA FISH for Detection of R. buchneri in Ticks

**Figure 7:**
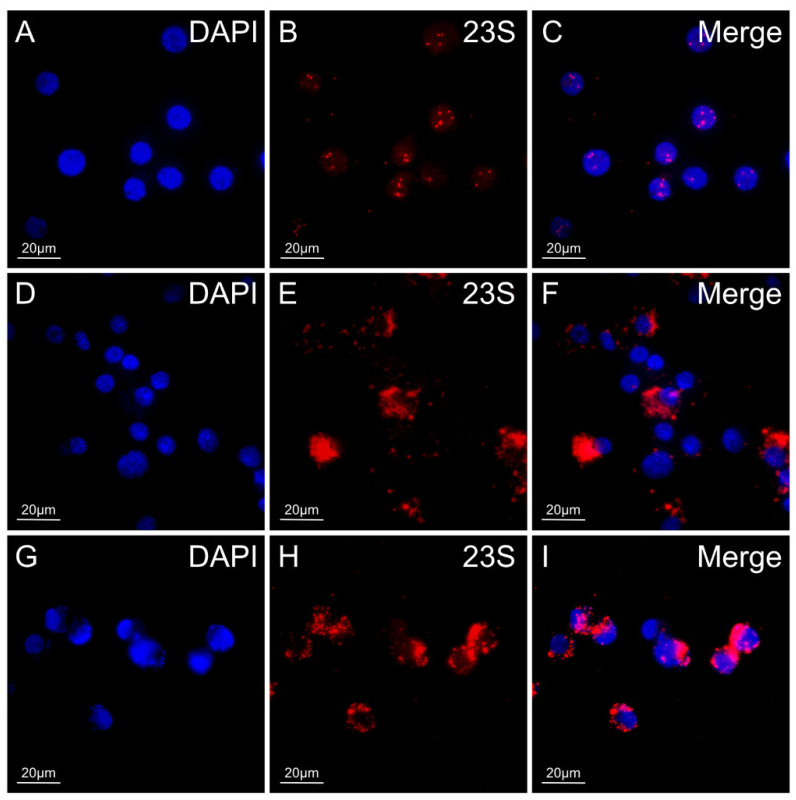
In vitro single-plex 23S Fast Red analysis of wildtype and transformant (GFPuv) *R. buchneri* grown in IRE11 tick cells fixed and labeled with the 23S rRNA Fast Red-labeled rickettsial probe (red). (**A**–**C**): Uninfected IRE11 cells. (**D**–**F**): IRE11 cells infected with wildtype *R. buchneri*. (**G**–**I**): IRE11 cells infected with transformed *R. buchneri*. Blue DAPI staining corresponds to the nuclei in all images. Merged signals (**C**,**F**,**I**) correspond to the combination of DAPI and TRITC (23S Fast Red) filters. All images were taken on an Olympus BX61 DSU confocal microscope at 60× objective magnification Size bars indicate 20 µm. No GFPuv signals are present in (**I**). Raw data images were edited with ImageJ Fiji software to reduce background fluorescence. The excitation and emission parameters were 4′,6-diamidino-2-phenylindole (DAPI): Excitation 350 nm, emission 470 nm; Tetramethylrhodamine (TRITC): Excitation 557 nm, emission 576 nm.

We developed a Fast Red-labeled 23S rRNA rickettsial probe for the in situ detection of rickettsiae in field-collected *I. scapularis* (Figure 7). The accuracy and specificity of the probe were checked against wildtype or GFPuv *R. buchneri* grown in IRE11. Control uninfected IRE11 cells exhibited minor non-specific binding of the 23S rickettsial probe to DAPI-stained nuclei (Figure 7A–C), which we attributed to insufficient washing and minor contamination. The probe exhibited a high degree of accuracy when applied to wildtype or transformed *R. buchneri* (Figure 1D–I), with strong fluorescent signals seen surrounding the nuclei of infected IRE11 cells. Additionally, the assay confirmed the GFPuv in transformed *R. buchneri* had no detrimental effects on the efficacy of the Fast Red-labeled 23S rickettsial probe. Taken together, the in vitro RNA FISH results validated the accuracy and specificity of the 23S rRNA Fast Red probe for detecting rickettsiae in tick cells.

**Figure 8:**
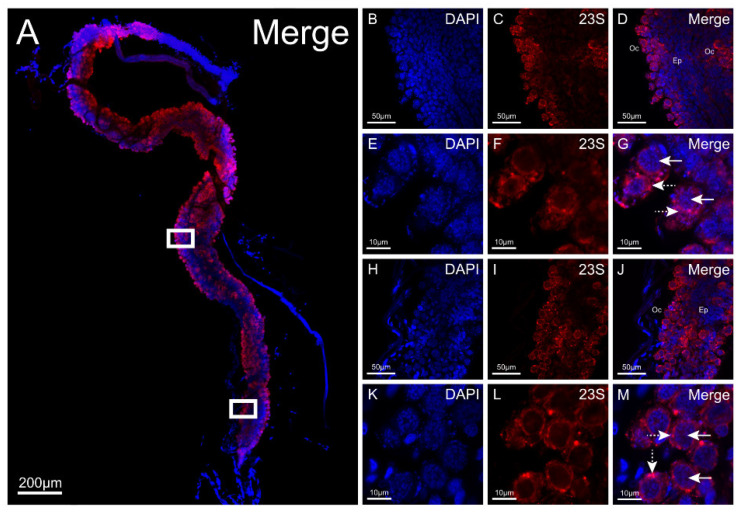
Low- and high-magnification images of a dissected whole ovary from a flat *I. scapularis* female utilizing a single-plex, Fast Red-labeled 23S rickettsial probe. (**A**): Low magnification (10× objective) of dissected whole ovary. White boxes in panel (**A**) indicate high-magnification (60× objective) scan areas. (**B**–**D**): 60× objective magnification images of the part of the ovary demarcated by the top white box. (**E**–**G**): Enlarged images of (**B**–**D**). (**H**–**J**): 60× objective magnification images of the part of ovary demarcated by the bottom white box. (**K**–**M**): Enlarged images of (**H**–**J**). Oc: Developing oocytes; Ep: Epithelial cells. Solid white arrows: DAPI-stained nuclei of developing oocytes; Dotted white arrows: *R. buchneri* located in the ooplasm surrounding nuclei of developing oocytes; Size bars indicate 200 µm (**A**); 50 µm (**B**–**D**,**H**–**J**); 10 µm (**E**–**G**,**K**–**M**). All images were taken with a Nikon A1 Spectral Confocal microscope utilizing combination of DAPI and TRITC (23S Fast Red) filters. Raw data images were edited with ImageJ Fiji software to reduce background fluorescence. The following excitation and emission parameters were utilized for fluorescent imaging; 4′,6-diamidino-2-phenylindole (DAPI): Excitation 350 nm, emission 470 nm; Tetramethylrhodamine (TRITC): Excitation 557 nm, emission 576 nm.

We used the probe to evaluate the tissue tropism of *R. buchneri* in *I. scapularis* that were collected at Camp Ripley, Minnesota. The FISH assay was performed on whole organs dissected from flat (unfed) females. Unfed females collected at this site contained an average of 10^7^ *R. buchneri gltA* (genome) copies [29]. We examined ovaries, seminal receptacles, salivary glands, midguts, and synganglia. At least 10 organs from each organ system were viewed using confocal microcopy over the course of three experimental replications. Low (4× or 10×) and high (60×) objective magnification images were taken for all examined organs. All ovaries dissected from flat females evaluated with the rickettsial probe exhibited strong red fluorescent signals throughout the entire organ (Figure 8A). At low magnification, *R. buchneri* was found to inhabit the developing oocytes along the entire outside edge of the organ. When examined using high magnification, *R. buchneri* was detected in the ooplasm surrounding the nucleus of the developing oocytes (Figure 8E–G,K–M).

There was no specific binding of the probe to salivary glands, seminal receptacles, midguts, or synganglia (not shown) when these tissues were examined with the single-plex 23S Fast Red-labeled rickettsial probe. Non-specific red fluorescent signals were seen throughout the salivary glands, a finding that has been previously reported when examining the salivary glands of *I. scapularis* [46]. However, these signals do not indicate positive *R. buchneri* detection when examined under high magnification due to the absence of colocalization signals of rickettsia-like bacteria observed under DAPI and 23S rRNA Fast Red filters. There was no DAPI signal indicating the absence of DNA associated with these signals. Additionally, fluorescent signals inside the salivary duct are observed, but again, co-localized signals of rickettsia-like bacteria were not detected using the DAPI and 23S Fast Red filters when viewed under high magnification. It is important to note that we observed this result in tracheal ducts throughout all evaluated organ systems. Evaluated seminal receptacles had an affinity for high levels of non-specific binding within the main body. In the midguts, red fluorescent signals were observed throughout the organ system. Strong red fluorescent signals were observed in globular structures in the distal end of the midgut lobe. We believe these structures to be representative of lipids. The absence of rickettsia-like bacteria using the DAPI filter when viewed under high magnification confirms there were no *R. buchneri* in the midgut. None of the examined synganglia exhibited positive fluorescent *R. buchneri* signals. Red fluorescence was also observed in tracheae surrounding the synganglion, but as mentioned previously, this phenomenon was frequently observed throughout all examined organ systems whenever tracheal-like structures were present and are likely products of non-specific binding.

## 4. Discussion

In striking contrast to its behavior in the cell line IRE11 from *I. ricinus* embryos, *R. buchneri* did not establish robust infections in *I. scapularis* cell lines ISE6 and ISE18, both also of embryonic origin [5]. Each cell line is relatively homogeneous when examined by light and electron microscopy. Despite this apparent morphological homogeneity, it is important to note that the lines were established using a primary culture of fragmented whole embryos and their tissue(s) of origin remains unknown. The cell line IRE11 is comprised of phagocytic cells, whereas ISE6 cells do not display this capability [47]. With its numerous mutations in genes involved with rickettsial invasion and motility [24], it is likely that *R. buchneri* is dependent on host cell intervention (phagocytosis) to enter and colonize prospective host cells. It is of interest to note that rickettsiae, possibly *R. buchneri*, have been observed in *I. scapularis* hemocytes [27]. In addition, the electron dense ellipsoidal granules and extensive filopodia observed in IRE11 cells are very similar to those seen in *I. ricinus* hemocytes [48,49].

The phenotypic differences between tick cell lines are poorly defined. Karyotype analyses indicate that individual cells, in terms of chromosome counts, in *Ixodes* cell lines vary and have increased or decreased chromosome numbers that deviate from the 2n = 28 for *Ixodes* ticks [50,51]. The impact of these chromosomal alterations on the functional genome and protein expression needs examination. Loginov et al., 2020 [52] compared IRE11, ISE6 and ISE18 lines using mass spectrometry (MS, MALDI-TOF), along with classical proteomic methods (2D electrophoresis) and principal component analysis with the MS profile tick organs (ovaries, salivary gland, gut, and Malpighian tubules). The MS comparison demonstrated there were different “organ-specific” proteins expressed by the cell lines, including those specific to ovaries, the gut, salivary glands, and Malpighian tubules. Each cell line had its distinctive profile and varied in organ-specific proteins expressed. These profiles may be a result of multiple cell types or indicate that we have selected for cell type(s) expressing several of these organ specific proteins. None of the lines have been cloned, and techniques for the clonal analysis of tick cell lines remain to be developed to answer this question.

Tick cell lines and wildtype or transformed symbionts are research tools for analyzing the interactions between rickettsiae and ticks under defined and controlled conditions. *Rickettsia buchneri* exhibits slow growth compared to pathogenic rickettsiae, with doubling times of multiple days in contrast to mere hours, further providing evidence for its classification as a non-pathogenic rickettsial endosymbiont. The growth of *R. buchneri* in *I. scapularis* takes place after a blood meal and while the blood meal is being digested [29]. The growth rate of *R. buchneri* during this phase is several days and mirrors the slow growth in IRE11 cells (this study). This suggests the growth of *R. buchneri* is tightly regulated or controlled, but the responsible mechanisms are unknown. When calculating the growth curve of the bacteria, qPCR was a more accurate method for quantification as it determined the number of genomic copies as opposed to measuring the GFPuv fluorescence intensity using the plate reader. The results obtained when incubating a multiwell plate at 30 °C indicated that GFPuv synthesis occurred at a slower rate than genome replication. Incubation at 30 °C is close to the upper limit for *R. buchneri* growth in vitro (32 °C) [25], which could explain the difference between the two methods. However, this is still lower than the skin surface temperature of laboratory mice [53], emphasizing that other parameters are also important in vivo, such as the length of exposure to higher temperatures, as larvae and nymphs feed for only a few days. Nevertheless, qPCR is costly and time intensive compared to analyzing growth characteristics of transformed bacteria with a fluorescence plate reader. While a growth curve determined via a fluorescence plate reader might not be as accurate compared to qPCR, it is still an effective method for characterizing the growth of *R. buchneri* under different environmental conditions.

The cell line IRE11 was used to validate the application of RNA FISH to detect *R. buchneri* in organs of *I*. *scapularis* ticks collected at Camp Ripley, Minnesota. However, due to the conserved nature of the 23S gene across rickettsiae, the probe could only be designed to identify rickettsiae at the genus level. The RNA FISH assay detected positive *R. buchneri* signals in the ooplasm of developing oocytes, but not in salivary glands, the seminal receptacle, midgut, or synganglion. The various compounds found in the salivary glands of ticks could have potentially interfered with the probe causing non-specific binding [54]. The strong positive signals of *R. buchneri* visualized in the developing oocytes of flat females further support the contemporary understanding of *R. buchneri* as a transovarially transmitted endosymbiont. It is plausible to infer *R. buchneri* plays a role in nutrient supplementation to the developing oocytes during oogenesis as its genome encodes pathways for essential B-vitamin synthesis [24]. Similarly, *R. buchneri* may play a role in supplementing nutrients to the tick host during long periods of starvation that are characteristic of the life cycle of *I. scapularis*, or to the nutrient-poor blood meal of a strictly hematophagous arthropod. Additionally, *R. buchneri* located within tick tissues could interfere with the infection and persistence of pathogens as the genome of *R. buchneri* has been shown to encode genes for antibiotic production [45]. Da Silva Costa et al. [55] analyzed the distribution of *R. rickettsii* in the ovarian cells of *Rhipicephalus sanguineus*. These authors confirmed the presence of *R. rickettsii* in the developing oocytes, mainly in the periphery surrounding the developing oocytes and cytoplasm of pedicel cells. This finding indicates the potential for both pathogenic and non-pathogenic rickettsiae to inhabit the oocytes of an Ixodid tick. Indeed, *Rickettsia peacockii*, a symbiont of a known vector of *R. rickettsii*, the Rocky Mountain wood tick, *Dermacentor andersoni,* exclusively infects the ovaries of female ticks and is passed on transovarially [56]. Ticks infected with *R. peacockii* are unable to maintain *R. rickettsii* between generations, suggesting a causal relationship [57]. This raises questions about *R. buchneri’s* ability to interfere with vertical transmission of pathogenic rickettsiae, which *I. scapularis* does not transmit. However, critical experiments investigating the ability of a rickettsial symbiont in *I. scapularis* to disrupt the transmission of pathogenic rickettsiae are lacking.

Al-Khafaji et al. [58] used ultrasensitive molecular methods to detect *R. buchneri* in the salivary glands, and other organs, of partially engorged *I. scapularis* females. The authors concluded that *R. buchneri* infect the salivary glands based on mass spectrometry, qPCR, and a FISH assay detecting *R. buchneri*-specific proteins, the *Rickettsia*-specific *gltA* gene, and a *Rickettsia*-specific fluorescently labelled DNA probe, respectively. The data demonstrate that the tissue tropism of *R. buchneri* is wider than acknowledged. There are few studies on the impact of *R. buchneri* on the transmission of pathogens by *I scapularis* and this needs to be further evaluated. Differences in the prevalence and quantity of *R. buchneri* in *I. scapularis* across geographic locations are well documented in previous studies [25,28,45,59,60,61,62,63]. Due to this variability, tissue localization of *R. buchneri* from different geographical locations should be examined further.

## 5. Conclusions

The sequestration of *R. buchneri* in the ovaries of female *I. scapularis*, its slow growth dynamics, and ability to grow high numbers in tick cells cultured in vitro strengthens the contemporary understanding of it being a transovarially transmitted, non-pathogenic endosymbiont. Its exact role and contribution to the biology of its tick host are not known, but its localization in tick ovaries facilitates transovarial maintenance. However, circumstantial evidence that it is responsible for preventing the colonization of black-legged ticks with pathogenic rickettsiae remains to be substantiated.
